# Editorial: ANCA-associated vasculitis treatment: outcomes and complications

**DOI:** 10.3389/fimmu.2025.1719890

**Published:** 2025-10-22

**Authors:** Matija Crnogorac, Lea Salamon, Maria Letizia Urban, Giacomo Emmi

**Affiliations:** ^1^ Department of Nephrology and Dialysis, Dubrava University Hospital, Zagreb, Croatia; ^2^ Department of Rheumatology and Immunology, Dubrava University Hospital, Zagreb, Croatia; ^3^ School of Medicine, University of Zagreb, Zagreb, Croatia; ^4^ Department of Medical, Surgical and Health Sciences, University of Trieste, Trieste, Italy; ^5^ Clinical Medicine and Rheumatology Unit, Cattinara University Hospital, Trieste, Italy

**Keywords:** ANCA, vasculitis, biomarkers, EGPA, cluster analysis

ANCA-associated vasculitis (AAV) is a group of rare, systemic, autoimmune diseases that are characterized by the inflammation of the small blood vessels and the usual presence of circulating ANCA antibodies (1). Thanks to advancements in treatment options, the prognosis for these diseases has significantly improved, and the risk of relapse has decreased (2). There is also an increased need to identify clinical subtypes within the classical AAV classification that would, based on individual disease traits and particular biomarkers, benefit from specific therapeutic approaches (3). Explored biomarkers might potentially allow for more personalized plans for patients’ retreatment by predicting disease activity or flares before clinical relapse (4).

The aim of this Research Topic is to describe the state of the art in AAV prognostic tools, treatment goals, and potential biomarker use. This Research Topic contains 5 articles, including two original research publications and three reviews, which nicely balance biomarker research with subtype clustering and specific treatment modalities, supporting the importance of this topic.

Moving beyond earlier classification systems of rare diseases is necessary even within specific clinical phenotypes in order to have better prognostic information, which could guide treatment decisions. In their multicenter cohort study, Okazaki et al. identified four unique subgroups of microscopic polyangiitis (MPA) patients with different outcomes. The two out these four subgroups, one with renal involvement and diffuse alveolar hemorrhage (DAH) and the other including elderly onset patients with systemic inflammation, had the worst overall survival, and the first one highest chance for ESKD. This suggests the importance of pooling data and conducting cluster analyses, as demonstrated in the works by the FAIRVASC consortium (5). An individualized treatment approach for each subgroup may be required in order to improve survival and reduce the risk of ESKD. (Okazaki et al.)

In their interesting review, Alberici et al. explored the challenges of defining treatment goals for AAV patients. Treatment advances in recent decades improved overall patient survival, reduced end-organ damage and turned AAVs into chronic diseases. The authors also noted that preserving patients’ quality of life has become as important as controlling disease activity. This requires the clinicians to balance both clinical and patient needs. One of the most important aspects is reducing treatment side effects by developing more targeted therapies. The future of successful management of AAV will require reducing AAV organ damage and achieving patient satisfaction (Federici A et al.).

Treatment goals often depend on assessing disease activity, for which tools are still limited. Therefore, there is a growing need for adequate and validated candidate biomarkers. On this note, Marvisi et al., in their detailed review, explored a variety of serological, cellular, and urinary biomarkers, with a focus on their use in assessing disease activity, disease-related damage, and prognosis. It is increasingly plausible that the future of personalized treatment and disease activity assessment will be based on biomarker panels rather than individual biomarkers. However, at the moment, according to the authors, there are two promising, commercially available biomarkers: urinary sCD163 and MCP-1 (6, 7). Both appear to distinguish well between active renal AAV and remission (Marvisi et al.).

Regarding eosinophilic granulomatosis with polyangiitis (EGPA), the original study by Shiomi et al. and the review of Lazzeroni et al. complement each other well. Both explored the role of biologics in treating EGPA.

In their REVEAL study, Shiomi et al. showed that mepolizumab contributes to both disease activity control and glucocorticoid (GC) dose reduction in their cohort of patients with EGPA, which may increase patient survival and reduce GC side effects.

This was further reaffirmed in a detailed review presented by (Lazzeroni et al.). The authors reviewed treatments consisting of benralizumab, mepolizumab, and reslizumab protocols. All of these anti-IL-5/IL-5 receptor drugs have been shown to be effective in remission control and corticosteroid tapering. The analyzed data strongly suggest the benefits of integrating anti IL-5/IL-5 receptor therapies into EGPA treatment strategies to both improve patient outcomes and reduce the side effects of prolonged GC therapy. However, the challenge remains in determining when to initiate anti-IL5/IL-5R therapy, as this has not been uniformly addressed across the included studies.

In conclusion, this Research Topic collects studies highlighting the current state of the art in the field of AAVs, together with original manuscripts identifying both treatment options and new ways of clustering AAVs. These contributions aim to advance prognostic tools and contribute to further progress in this field. We believe that readers will gain a better understanding of this topic (Shiomi et al.) (Lazzeroni et al.).

## References

[1] Jennette JC Falk RJ Bacon PA Basu N Cid MC Ferrario F. Revised international chapel hill consensus conference nomenclature of vasculitides. Arthritis Rheumatol. (2012) 65:1–11. doi: 10.1002/art.37715, PMID: 23045170

[2] Alberici F Jayne DRW. Impact of rituximab trials on the treatment of ANCA-associated vasculitis. Nephrol Dial Transplant Off Publ Eur Dial Transpl Assoc Eur Ren Assoc. (2014) 29:1151–9. doi: 10.1093/ndt/gft318, PMID: 24126571

[3] Tedesco M Gallieni M Pellegata F Cozzolino M Alberici F. Update on ANCA-associated vasculitis: from biomarkers to therapy. J Nephrol. (2019) 32:871–82. doi: 10.1007/s40620-019-00628-9, PMID: 31301058

[4] Charles P Terrier B Perrodeau É Cohen P Faguer S Huart A. Comparison of individually tailored versus fixed-schedule rituximab regimen to maintain ANCA-associated vasculitis remission: results of a multicentre, randomised controlled, phase III trial (MAINRITSAN2). Ann Rheum Dis. (2018) 77:1143–9. doi: 10.1136/annrheumdis-2017-212878, PMID: 29695500

[5] Gisslander K White A Aslett L Hrušková Z Lamprecht P Musiał J. Data-driven subclassification of ANCA-associated vasculitis: model-based clustering of a federated international cohort. Lancet Rheumatol. (2024) 6:e762–70., PMID: 39182506 10.1016/S2665-9913(24)00187-5

[6] Moran SM Scott J Clarkson MR Conlon N Dunne J Griffin MD. The clinical application of urine soluble CD163 in ANCA-associated vasculitis. J Am Soc Nephrol. (2021) 32:2920–32. doi: 10.1681/ASN.2021030382, PMID: 34518279 PMC8806104

[7] Kronbichler A Kerschbaum J Gründlinger G Leierer J Mayer G Rudnicki M. Evaluation and validation of biomarkers in granulomatosis with polyangiitis and microscopic polyangiitis. Nephrol Dial Transplant. (2016) 31:930–6. doi: 10.1093/NDT/GFV336, PMID: 26410887

